# Evaluating the Sequelae of Mastoidectomy for Acute Mastoiditis: A Long-Term Follow-Up Study of Mastoid Function

**DOI:** 10.3390/jcm14196689

**Published:** 2025-09-23

**Authors:** Matija Švagan

**Affiliations:** Department of Otorhinolaryngology, University Medical Center Maribor, 2000 Maribor, Slovenia; matija.svagan@icloud.com

**Keywords:** acute mastoiditis, mastoidectomy, long-term outcomes, pediatric otorhinolaryngology

## Abstract

**Background:** Despite the widespread use of antibiotics, acute mastoiditis (AM) and related complications resulting from acute purulent otitis media continue to occur, predominantly in children. Although numerous studies have focused on the pathogenesis, aetiological agents, and treatment of AM, comprehensive investigations of the long-term outcomes of AM and the physiological consequences of surgical intervention in the temporal bone are lacking. **Methods:** Thirty patients who had undergone mastoidectomy for acute mastoiditis at a median age of 2.12 years were invited for evaluation at least five years postoperatively. The assessment included the Chronic Otitis Media Questionnaire 12, clinical examination with otomicroscopy, extended high-frequency pure-tone audiometry, distortion product otoacoustic emissions, middle ear impedance testing, and a newly developed protocol for noninvasive mastoid function measurement. Results were compared with a control group of 30 ears and with a group of 30 ears treated solely with tympanostomy for acute otitis media at risk of mastoiditis. **Results:** Although mean Chronic Otitis Media Questionnaire 12 scores were below 1 point, patients who had undergone mastoidectomy reported slightly greater difficulties with hearing in both quiet and noisy environments, along with an increased perception of tinnitus and unpleasant sensations around the ear. Otomicroscopy revealed minor structural changes in the test groups, which were absent from the control group. Pure-tone audiometry demonstrated approximately 10 dB higher thresholds at high and extended high frequencies, with similar findings observed in the distortion product otoacoustic emissions. Middle ear impedance testing indicated elevated stapedius reflex thresholds in the mastoidectomy group, while other parameters showed no statistically significant differences. Mastoid function testing demonstrated preserved pressure-buffering capacity but reduced thermal insulation of the vestibular organ under extreme thermal stimulation—an occurrence rarely encountered in daily life. **Conclusions:** In the long term, most patients recovering from acute mastoiditis exhibit only minor functional and structural sequelae, and the impact of mastoidectomy appears negligible compared with less invasive surgical interventions.

## 1. Introduction

Despite the widespread use of antibiotics, acute mastoiditis (AM) and its associated complications—typically arising from acute suppurative otitis media (AOM)—continue to occur, predominantly affecting children. Although their incidence has significantly declined due to improved treatment strategies, surgical intervention remains a central component of management, particularly in cases involving intracranial complications and microbial resistance [1,2,3,4,5,6,7].

In recent years, there has been a notable increase in studies investigating more conservative approaches to managing uncomplicated AM, either by minimizing surgical intervention, such as mastoidectomy, or by entirely avoiding surgery. These studies report commendable recovery outcomes; nevertheless, the trend toward reducing the frequency and extent of surgical drainage procedures raises significant concerns. Potential issues include an increased risk of severe complications, extended duration of treatment, greater reliance on antibiotic therapy, and diminished surgical proficiency and expertise [1,3,4,8,9,10,11,12,13].

While numerous studies have examined the pathogenesis, microbial etiology, and short-term treatment outcomes of acute mastoiditis, few have addressed its long-term sequelae related to inflammatory disorders and oxidative stress [14]. However, the physiological consequences of surgical intervention involving the mastoid cell system [10,15,16] have not been adequately investigated. To address this gap, we conducted a comprehensive study to evaluate the long-term effects of surgically treated acute mastoiditis and, in particular, to assess the impact of mastoidectomy on ear function. We employed a range of clinically available and validated noninvasive methods, including the Chronic Otitis Media Questionnaire-12 (COMQ-12), extended high-frequency pure-tone audiometry, distortion product otoacoustic emissions (DPOAE), and noninvasive mastoid function testing.

## 2. Materials and Methods

### 2.1. Patient Selection

A retrospective review was conducted of medical records of pediatric patients who underwent mastoidectomy for AM between July 2001 and October 2019 at the Department of Otorhinolaryngology, University Clinical Center Maribor. During this 18-year period, the surgical indications for mastoidectomy remained consistent and included at least two of the following clinical signs: otalgia, fever, mastoid pain or tenderness on palpation, postauricular swelling and erythema, auricular protrusion, and sagging of the ear canal (suggestive of an abscess in the ear canal). All procedures were performed under general anesthesia by the same group of surgeons, following a standardized surgical protocol as soon as an otologic surgeon was available. The technique involved a postauricular incision, drilling of the cortical mastoid bone, opening of the major air cell system, and exposure of the epitympanum. Tympanostomy tubes were inserted for middle ear drainage, and a drainage tube was placed in the mastoid cavity. The surgical site was closed in two layers. Inclusion criteria required that patients undergo mastoidectomy with tympanostomy in one ear for acute mastoiditis and tympanostomy in the contralateral ear for concurrent fulminant acute otitis media. The contralateral tympanostomy was an essential criterion, as this allowed within-subject comparison and helped to minimize limitations inherent to retrospective studies (e.g., interindividual variability, differing symptom duration, and pathogen diversity).

Out of 109 patients identified, contact information was available for 97, and 67 met the additional inclusion criteria. Of these, 30 consented to participate in the study.

Thirty healthy volunteers, matched to the test cohort by age, without documented or anamnestic history of ear infections, hearing impairment, or balance disorders, formed the control group. Volunteers with responses exceeding 0 points on any of the COMQ-12 item were excluded.

For analysis, data were divided by ear, forming three groups: tympanostomy and mastoidectomy group (Group TM—30 ears), tympanostomy-only group (Group T—30 ears), and control group (Group C—30 ears).

### 2.2. Data Collection

The study was conducted from 1 September 2022, to 30 March 2024, at the Department of Otorhinolaryngology, University Clinical Center of Maribor. The sample size was limited by volunteer response rates from the test group, predominantly adolescents and young adults typically without residual symptoms. The relatively low incidence of AM further constrained participant numbers. Nevertheless, the sample size aligns with comparable studies on AM [17,18,19].

### 2.3. Review of Medical Records and Examination

Medical records were reviewed for demographic data, preoperative symptoms, intraoperative findings, diagnostic methods, laboratory parameters, microbiological findings, and postoperative complications. Otomicroscopy was conducted using a standard office microscope, employing a grading system with a Likert scale (0–3) for pathology evaluation, including tympanic membrane atrophy, thickness, scarring, myringosclerosis, and myringitis. Retraction severity was graded from 0 to 4 according to Toš and Sadé classifications [20,21].

### 2.4. Sensory Testing

**Figure 1:**
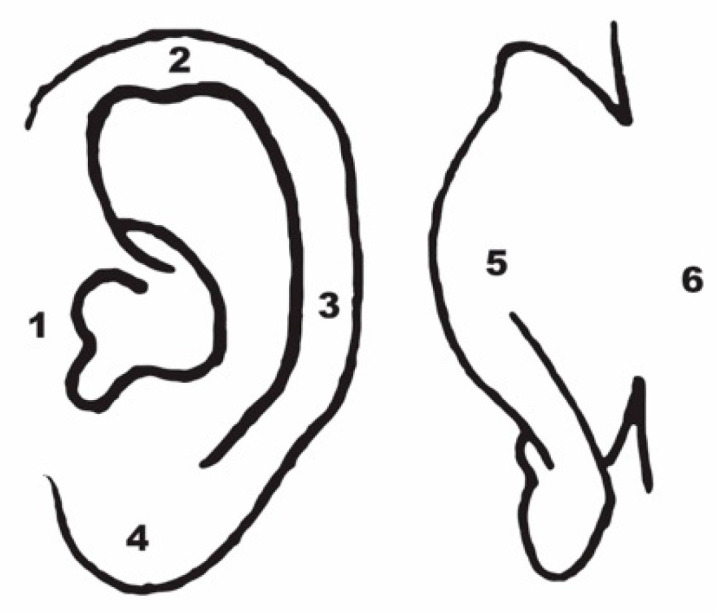
Sensory testing locations.

Auricular sensory testing was performed using a 1.65 Semmes-Weinstein monofilament at six predefined auricular locations (Figure 1) [22]. One point was assigned for each location at which touch was detected by the participant.

### 2.5. Chronic Otitis Media Questionnaire 12

Participants completed the COMQ-12, a validated 12-item, disease-specific health-related quality-of-life questionnaire with Likert-scale responses (0–5) [23,24]. The COMQ-12 questionnaire was selected to identify persistent or chronic ear-related symptoms, although chronic ear problems were not anticipated in the study population.

### 2.6. Audiometry

Extended high-frequency pure tone audiometry (125 Hz–20 kHz) was conducted using an Interacoustics AC40 Clinical audiometer (Middelfart, Denmark). Audiometric results were categorized into low-tone average (LTA, <500 Hz), middle-tone average (MTA, 0.5–4 kHz), high-tone average (HTA, 4–8 kHz), and extended high-tone average (EHTA, 8–20 kHz). For calculation of MTA same method was used as per widely known pure-tone average (PTA) [25].

### 2.7. Middle Ear Impedance Testing

Single-band (256 Hz) tympanometry and stapedial reflex thresholds (500, 1000, 2000, 4000 Hz) were measured with an Interacoustics AT235 tympanometer (Middelfart, Denmark). Wide-band tympanometry was conducted using Interacoustics Titan hardware (Middelfart, Denmark), recording external auditory canal volume, tympanometric peak pressure (TPP), compliance, and middle-ear resonant frequency.

### 2.8. Distortion-Product Otoacoustic Emissions

DPAOEs were recorded using Interacoustics Eclipse equipment (Middelfart, Denmark), with stimulus levels of 65 dB (f1) and 55 dB (f2), an f2/f1 ratio of 1.22, test duration of 60 s, and minimum reliability of 98%. Measurements were conducted twice across frequencies ranging from 500 Hz to 10 kHz. Only reliable emissions were included, averaged, and frequency-averaged similarly to audiometry.

### 2.9. Ultrasound Examination

**Figure 2:**
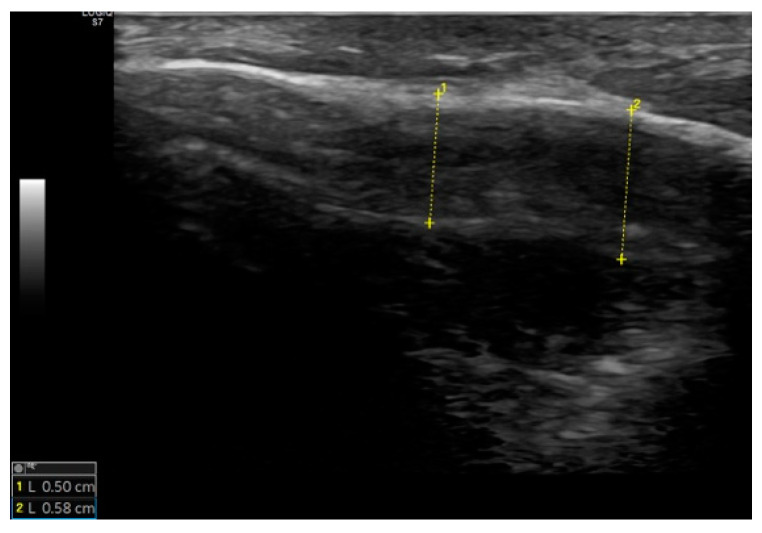
Measurements of thickness of the mastoid cortex. Dashed yellow lines represent the software measuring tool on the mastoid cortex.

Ultrasound is a non-invasive imaging method that allows examination of deeper tissues. While typically used for soft tissue assessment, it is also effective for examining the periosteum and cortical bone. Using ultrasound, we can identify potential defects in the mastoid bone and periosteum post-surgery, as well as measure the thickness of the skin over the mastoid and cortical bone (Figure 2). To ensure consistency and minimize interobserver variability, all measurements were performed by the same examiner, an experienced head and neck ultrasonographer. For each parameter, five measurements were obtained and the mean value was calculated.

### 2.10. Non-Invasive Assessment of Mastoid Function

The two mastoid functions that can be evaluated non-invasively are its role as a pressure buffer and its thermal insulation capacity. For this purpose, we developed a reproducible protocol using standard clinical equipment. Cooling and warming of the skin over the mastoid are known to influence tympanometric peak pressure (TPP) and vestibulo-ocular reflex [26]. While TPP changes are easily quantifiable, nystagmus measurement is more technically demanding; therefore, video head impulse test (vHIT) was chosen for vestibulo-ocular reflex gain (VORg) assessment as it allows reliable statistical analysis under standard outpatient conditions.

Cooling is expected to reduce TPP. Assuming the mastoid behaves as a closed air-filled system, a temperature drop of 1 °C from normal body temperature would reduce middle ear pressure by approximately 32 daPa. In reality, the mastoid is neither closed (due to the Eustachian tube) nor of constant volume, as mucosal thickness changes [27]. Cooling likely reduces mucosal thickness through vasoconstriction of small perforating vessels in the cortical bone, increasing the air-filled volume and further lowering middle ear pressure. Considering these mechanisms, a total pressure drop of 50–80 daPa can be expected [26,28].

For VORg, no prior reference exists for temperature-induced change. Our experimental setup—subjects seated with horizontal alignment of the lateral semicircular canals—minimizes endolymph convection effects, suggesting that increased VORg is due to direct thermal effects on hair cells [29,30,31,32,33,34,35]. Our preliminary trials indicated an approximate 10% increase in VORg during cooling. In the context of mastoid thermal insulation, this response likely reflects the relationship between mastoid cell surface area and its volume.

Thermal conduction properties of overlying tissues are critical for interpreting these measurements. Reported conductivity values are ~0.545 W/mK for skin and ~0.68 W/mK for cortical bone; mastoid cellular bone is likely lower (~0.42 W/mK) [36,37,38,39]. Hydrogel pads, with high heat capacity, provide reproducible cooling to deeper structures. Middle ear temperature change is estimated indirectly by infrared tympanic thermometry, targeting the scutum region. Anatomical studies show the lateral semicircular canal lies at an average depth of ~12 mm from the mastoid surface, comparable to the distance from the surface to the scutum [40,41]. Given similar tissue conductivity, heat transfer should reach both sites simultaneously, allowing indirect assessment of mastoid temperature change at the depth of the lateral semicircular canal.

At the start, baseline measurements are taken for mastoid skin temperature, ear canal temperature, TPP, and VORg for the lateral semicircular canal. A hydrogel pad (5 × 10 × 2 cm, folded in half) cooled to –5 °C is placed over the mastoid process, directly behind the ear, with the auricle and skin protected by a four-layer cotton pad. Cooling is applied for 5 min, after which all measurements are repeated. Measurements can be completed within 1 min, after which the cooled hydrogel pad is reapplied for another 5 min. A third series of measurements is then performed. The total protocol duration is 12 min. The protocol is based on the experimental design described by Magnuson (2003) but differs in that VORg is used instead of nystagmography [26]. To ensure consistency and minimize interobserver variability, all measurements were performed by the same examiner using the same equipment. For VORg assessment, at least 40 reflex responses were collected for each participant.

### 2.11. Data Analysis

The statistical analysis was performed using the statistical package SPSS V29. Descriptive statistical methods were used for sample description. Results are presented as median values with 95% confidence intervals or frequencies and percentages. The Shapiro–Wilk test was used to confirm non-normal data distribution. Comparisons between groups were analyzed using two-sided Chi-square (χ^2^) tests, Kruskal–Wallis Tests, Independent Samples Median Tests (ISMT), Spearman Correlation and Mann–Whitney U test (MWU). Results were considered statistically significant for *p*-values below 0.05

## 3. Results

### 3.1. Characteristics of Patients Undergoing Mastoidectomy

A review of hospital electronic medical records from the University Clinical Center Maribor for the years 2009–2019 identified 109 patients meeting the inclusion criteria. None of these patients had regular follow-up visits at the Department of Otorhinolaryngology and Head and Neck Surgery. Contact information was available for 97 patients, of whom 67 fulfilled additional inclusion criteria and were invited to participate. Thirty patients responded to the invitation and completed the research protocol.

A single episode of uncomplicated AOM preceding AM was documented in 10% (3) of patients, with one patient (3.3%) experiencing two episodes. The remaining patients had no documented history of ear infections. Parents did not report pre-existing hearing loss before AM onset. Additionally, 96.7% (n = 29) of patients successfully passed neonatal hearing screening. The median age at surgery was 2.12 years (Q1: 1.24; Q3: 4.08), with the youngest patient being 0.64 years old and the oldest 11.33 years old. The left side was operated in 63.3% (n = 19) of cases, and 56.7% (n = 17) were male. The median time from symptom onset to surgery was 3.0 (Q1: 1.75; Q3: 6.25) days. Median hospital stay was 10.5 days (Q1: 8.0; Q3: 14.25), ranging from 5 to 78 days. The retroauricular drainage tube remained in place for a median of 9.5 days (Q1: 6.75; Q3: 12.0), ranging from 4 to 20 days.

Imaging (CT scan) was performed in 16.7% (n = 5) of cases. Median leukocyte count was 14.25 × 10^9^/L (Q1: 11.55; Q3: 20.05), ranging from 2 to 35 × 10^9^/L. Median C-reactive protein was 99.0 mg/L (Q1: 47.75; Q3: 160.75), ranging from 5 to 280 mg/L. Streptococcus pneumoniae was the predominant pathogen, isolated in 56.7% (n = 17) of cases. Streptococcus pyogenes group A was found in 20% (n = 6), while Turicella otitidis, Pseudomonas aeruginosa, and Staphylococcus aureus each appeared in 3.3% (n = 1) of cases. Sterile cultures were obtained in 10% (n = 3). Bilateral swabs yielded identical pathogens in 96% (n = 26) of cases. No surgical complications were associated with mastoidectomy. 30% (n = 9) of the patients received systemic antibiotic treatment prior to surgery; however, data regarding whether the administration was intravenous or oral were not available. Meningitis occurred in one patient, recurrent AOM in 20% (n = 6), and recurrent mastoiditis in 3.3% (n = 1). Among all 109 mastoidectomies performed at the department in the observed years, complications included labyrinthitis (4.5%, n = 5), meningitis (2.8%, n = 3), facial nerve paresis (2.8%, n = 3), sigmoid sinus thrombosis (1.8%, n = 2), sepsis (1.8%, n = 2), and posterior cranial fossa skull base defect (0.9%, n = 1). Surgical complications were rare, comprising one case each of excessive bleeding due to undiagnosed coagulopathy and postoperative retroauricular skin necrosis.

Median duration of routine follow-up was 13 months (Q1: 11; Q3: 16.25), ranging from 7 to 37 months. During this period, recurrent bilateral AOM occurred in 20% (n = 6) of patients, and repeat bilateral tympanostomy was necessary in 10% (n = 3). One patient (3%) developed tympanic membrane perforation post-AM, successfully managed with myringoplasty. Patients reported no ongoing issues or long-term hearing impairment. The interval from surgery to research protocol implementation was a median of 11.6 years (Q1: 6.83; Q3: 13.63), ranging from 4.98 to 22.07 years.

The overall median age at the time of protocol completion was 14.3 years (Q1: 11.21; Q3:16.25), ranging from 6.63 to 24.85 years, median age in Group TM was 14.1 years (Q1: 10.71; Q3: 15.76), in Group T 14.1 years (Q1: 10.71; Q3: 15.76), and in Group C 14.45 years (Q1: 12.05; Q3: 20.73).

### 3.2. Chronic Otitis Media Questionnaire 12

COMQ-12 questionnaire results are presented in Table 1. Mean scores per item were also reported for clarity and revealed very low values, all below 1. Statistically significant differences between groups were found for items 3, 4, 5, 6, and 7.

### 3.3. Tactile Sensitivity Testing of the Auricle

Reduced tactile sensitivity was observed in 10% (n = 3) of patients at location 5, in 6.6% (n = 2) at location 6, and in 3.3% (n = 1) at location 4. No sensory deficits were observed at other sites, in the control group, or in the contralateral ears (Figure 1).

### 3.4. Otomicroscopic Examination

Otoscopy findings are summarized in Table 2, including both median and mean values. No statistically significant differences were found between Groups TM and T. However, both differed significantly from Group C in the presence of tympanic membrane atrophy, myringosclerosis, and epitympanic retraction (Toš classification). No significant differences were observed for signs of active chronic otitis (myringitis) or conditions potentially affecting sound transmission (thickening, scarring, Sadé retraction).

### 3.5. Extended High-Frequency Pure-Tone Audiometry

**Figure 3:**
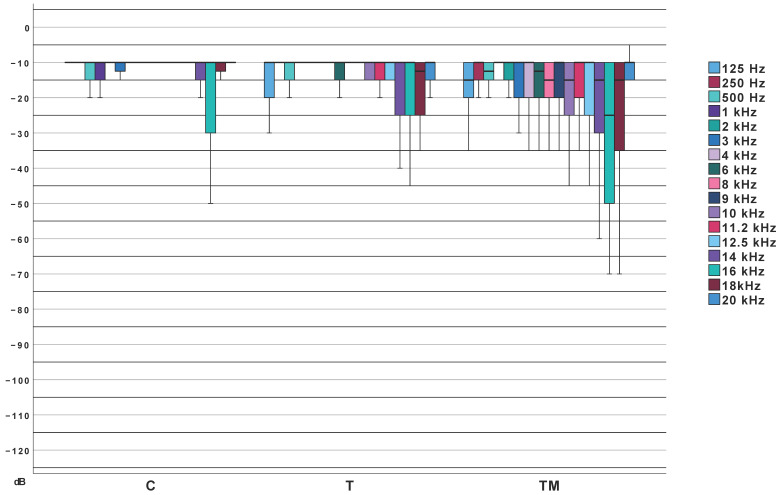
Median values of pure-tone audiometry thresholds. Clusters are presented in a format analogous to a clinical audiogram, frequencies are color coded; TM, T, C—groups.

**Figure 4:**
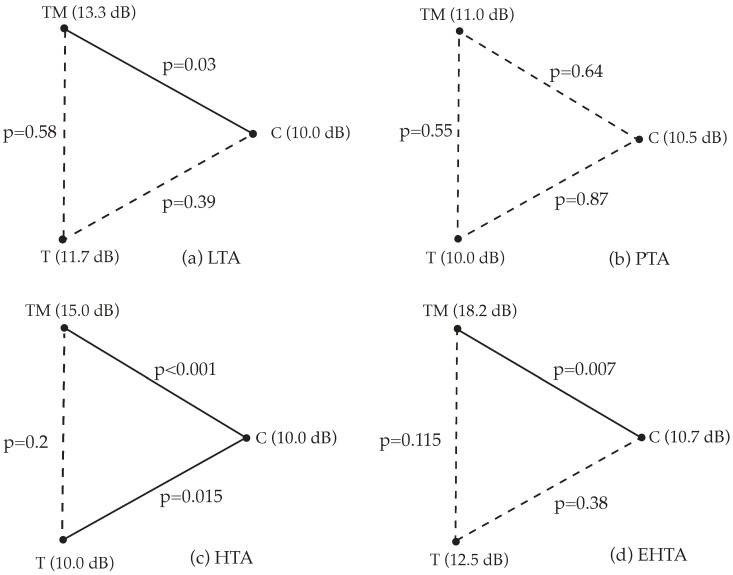
Direct comparison of pure-tone audiometry results (ISMT): (**a**) low-frequency average; (**b**) mid-frequency average; (**c**) high-frequency average; (**d**) extended high-frequency average; *p*—statistical significance; T, TM, C—groups.

Median threshold values are provided in Table 3. Statistically significant differences were identified in the low-, high-, and extended high-frequency averages. However, differences in mid-frequency averages (MTA/PTA) were not statistically significant. Figure 3 illustrates these results in an audiogram-style format. Pairwise comparisons showed: no significant differences between Groups TM and T across frequency ranges, significant difference between Groups T and C only in high-frequency averages and significant differences between Groups TM and C across all frequencies except mid-frequencies as visualized in Figure 4.

EHTA values significantly correlated with COMQ-12 responses regarding hearing in quiet (ρ = 0.480, *p* = 0.037), hearing in noise (ρ = 0.424, *p* = 0.019), and tinnitus perception (ρ = 0.392, *p* = 0.032). EHTA also correlated with the number of symptomatic days before surgery (ρ = 0.413, *p* = 0.023), with a near-significant correlation observed for HTA (ρ = 0.334, *p* = 0.071). Number of symptomatic days also significantly correlated with perceived hearing loss in noise (ρ = 0.384, *p* = 0.036).

### 3.6. Middle Ear Impedance Testing

Single- and wide-band tympanometry showed that Group C had significantly larger external auditory canal volumes (χ^2^ = 14.6; *p* < 0.001) than Groups TM and T. No other tympanometric measures showed significant intergroup differences.

**Figure 5:**
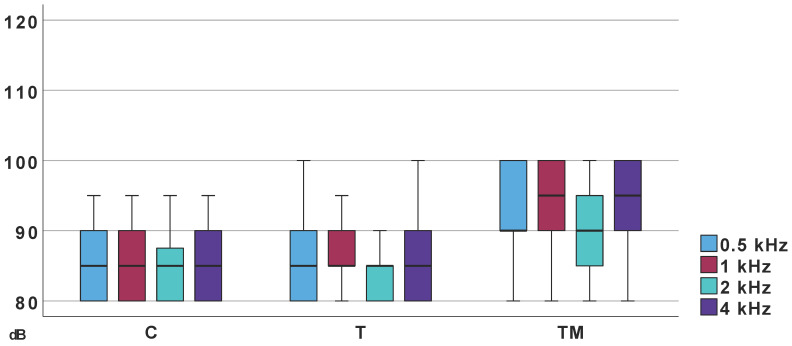
Median values of acoustic reflex threshold; T, TM, C—groups.

Ipsilateral acoustic reflex thresholds differed significantly across frequencies and groups. Significant differences were observed between Groups K and TM, and T and TM. No significant differences were found between Groups T and C (Figure 5).

### 3.7. Distortion Product Otoacoustic Emissions

**Figure 6:**
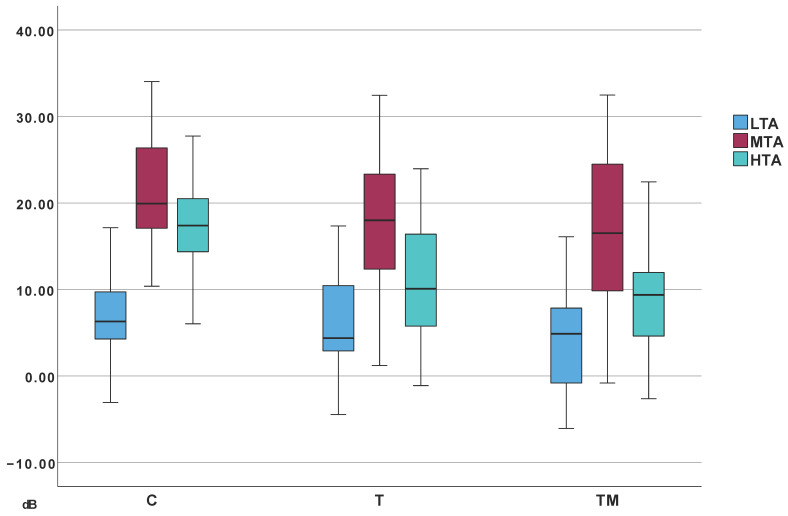
Median DPOAE amplitudes: LTA—low-frequency average, MTA—mid-frequency average, HTA—high-frequency average; T, TM, C—groups.

Median DPOAE levels corrected for noise are listed in Table 4. Frequency band averages were computed similar to audiometry: low tone average (LTA; 500 Hz), middle tone average (MTA; 1–4 kHz), and high tone average (HTA; 5–10 kHz). Results are visualized in Figure 6. Corrected DPOAE values significantly correlated with audiometric thresholds: Low-frequency: ρ = −0.279, *p* < 0.007; mid-frequency: ρ = −0.387, *p* < 0.001; high-frequency: ρ = −0.640, *p* < 0.001.

**Figure 7:**
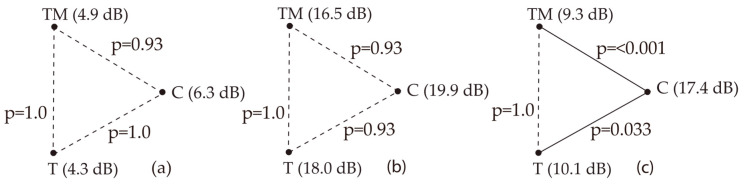
Direct comparison of DPOAE results (ISMT): (**a**) low-frequency average, (**b**) mid-frequency average, (**c**) high-frequency average; *p*—statistical significance; T, TM, C—groups.

Direct group comparisons showed no significant differences in low and mid-frequency DPOAE values. Significant differences at high frequencies were observed between Group C and both Groups T and TM (Figure 7).

### 3.8. Ultrasound Examination of the Mastoid

Ultrasound confirmed complete cortical healing in 96.7% (n = 29) of patients. One patient had a defect involving less than 25% of the lateral cortical surface. No significant differences were found between Groups K and TM in cortical bone or skin thickness over the mastoid (Table 5).

### 3.9. Mastoid Function Testing

Temperature values (mastoid and ear canal), VORg, and TPP are summarized in Table 6. To validate the non-invasive mastoid function test, we hypothesized that post-cooling values would significantly differ from baseline. All values except mastoid temperature change from 6 to 12 min met this criterion.

**Figure 8:**
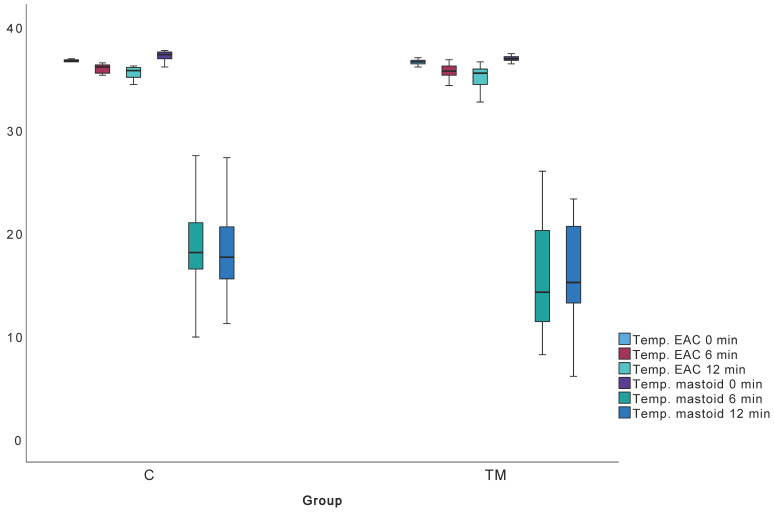
Measurements of ear canal temperature and mastoid skin temperature; TM, C—groups.

Mastoid skin temperature measurements significantly differed between Groups K and TM at all time points. Ear canal temperature differed significantly only at minute 0 (Figure 8). No significant differences were found in ear canal temperature changes (delta) over time. Mastoid skin temperature showed greater reduction in Group TM between 0–6 and 0–12 min (Table 7, Figure 8).

**Figure 9:**
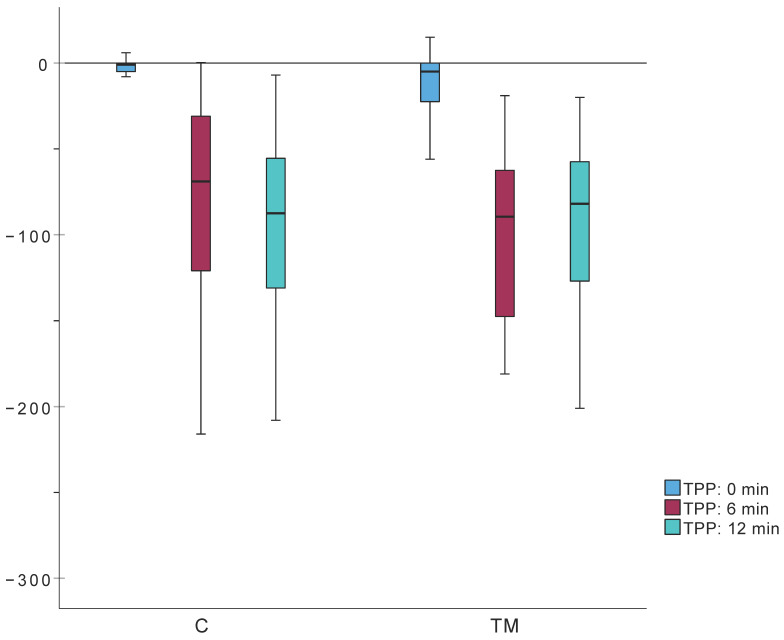
TPP measurements according to the protocol for non-invasive mastoid function assessment; TM, C—groups.

TPP values and changes did not significantly differ between groups (Table 7, Figure 9).

**Figure 10:**
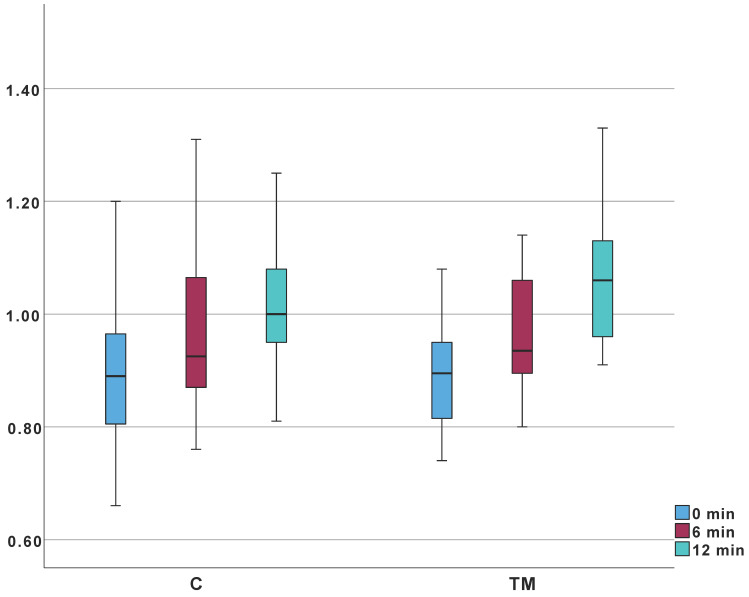
VOR gain measurements according to the protocol for non-invasive mastoid function assessment; TM, C—groups.

Absolute oVOR values showed no significant intergroup differences. However, the change from baseline to minute 12 was significantly different (*p* = 0.029) (Table 7, Figure 10).

Two participants discontinued the protocol at 6 min due to dizziness, nausea, and nystagmus. Both had reported no prior vertigo on the COMQ-12. No abnormalities were found in the patient with incomplete cortical healing on ultrasound.

## 4. Discussion

This study is unique in examining the sequelae of mastoidectomy performed in the context of AM treatment. To our knowledge, no previous research has investigated the long-term consequences of AM and its treatment in such detail.

While the incidence of AM and other complications associated with AOM is declining, this trend directly correlates with antibiotic usage [42,43]. Traditionally, mastoidectomy has been recognized as the most effective and reliable method for treating AM but is also the most invasive [18,44]. Increased antibiotic treatment efficacy has prompted numerous studies demonstrating that less invasive methods can effectively manage AM [2,4,5,10,15,17,19,45,46,47]. The question arises whether mastoidectomy remains necessary in contemporary AM treatment if recovery is achievable without surgery. Upon reviewing various treatment strategies, the broad definition of AM explains the variety in therapeutic approaches. However, available studies do not suggest that less invasive methods were exclusively used for milder AM forms; rather, mastoidectomy was predominantly employed when the efficacy of other methods was uncertain [4,10,11,19]. To advocate mastoidectomy as a viable treatment option in modern management of AM, our research must demonstrate comparability, effectiveness, safety, and minimal postoperative consequences. Researching AM inherently faces limitations, as it typically affects severely ill or life-threatened children, making prospective studies nearly impossible. Retrospective studies may inherently contain bias due to challenges in accurately assessing the severity of inflammation, pre-existing hearing loss, or subsequent hearing impairments.

Our study compared patients with concurrent bilateral fulminant AOM. One ear progressed to AM, while the other exhibited severe AOM without fulfilling AM criteria but showed potential for progression (53% had a lowered postero-superior wall of the external auditory canal, and all had severely lateralized eardrums). Since middle ear and mastoid cavities are interconnected, mastoid inflammation (infection) likely occurs during AOM episodes [48]. On the AM-affected side, patients underwent combined mastoidectomy and tympanostomy, whereas the contralateral ear received tympanostomy alone. Pathogens were identical bilaterally in 96% of cases, indicating similar infection severity and timing, differing only in mastoid intervention. It is important to note that inflammation was likely more severe in ears undergoing mastoidectomy, possibly attributing more sequelae to surgical intervention inadvertently. Given minimal prior AOM episodes and appropriate management of recurrent AOM, previous or subsequent infections were unlikely to significantly affect ear function.

We acknowledge the possibility of selection bias, as only 30 of the 67 eligible patients responded to our invitation. Although the motivation for participation is unknown, the demographics, inflammatory markers, microbiological profiles, length of hospital stay, and complication rates of our cohort were comparable to those reported in similar studies, supporting the assumption that the sample may be representative [15,17,18,19,49]. A longer hospital stay noted in our cohort reflects institutional practice, as patients remained formally admitted during acute phases despite outpatient management. Actual hospital duration correlates closely with drainage tube duration, comparable to other studies.

The median age at surgery (2.12 years) indicates limited pure-tone audiometry feasibility, while brainstem auditory potentials testing is typically unnecessary for normal-hearing children. Pre-AM hearing concerns were absent from parental reports, with no post-AM trauma or serious infections documented. Neonatal hearing screening, successful in 97%, further supporting comparability with larger studies and adequately limiting bias, thus validating our results’ applicability.

Analysis of AM treatment strategies emphasizes individualized decisions and the lack of standardized protocols [49,50,51]. Literature reviews consistently demonstrate mastoidectomy’s effectiveness, despite differing opinions regarding recurrence prevention [52,53]. Our cohort experienced minimal recurrence (3.3%), confirming mastoidectomy as highly effective, aligning with previous findings.

Mastoidectomy, a fundamental otologic procedure, carries complication rates between 1 and 3% [54], significantly lower in primary surgeries [55]. Our analysis revealed low complication rates (1.8%), all reversible, performed consistently by three surgeons. General anesthesia in children with upper respiratory infections poses significantly higher risk (up to 30%) [56,57]. Combining literature and our findings, mastoidectomy emerges as safe when performed by experienced surgeons, with anesthesia presenting primary risks.

Median audiometric thresholds demonstrated minor hearing loss (<10 dB) across all frequencies in both groups T and TM compared to controls. Despite this minimal loss, speech comprehension remained unaffected, as confirmed by the COMQ-12 questionnaire results. Detailed audiometric analysis revealed statistically significant differences between both tested groups and the control group, primarily within high-frequency ranges. Importantly, no statistically significant differences were observed in frequency averages between groups T and TM, indicating that the ears differed only in the performance of mastoidectomy on one side. This finding suggests that the direct impact of mastoidectomy on auditory function is minimal or negligible. Furthermore, it is likely that inflammation was more severe and prolonged in ears undergoing mastoidectomy, thus increasing the risk of inner ear damage. From this perspective, the influence of mastoidectomy on hearing outcomes might even be overestimated. Additionally, patients who underwent mastoidectomy earlier during the disease course showed better hearing outcomes, implying that timely mastoidectomy could contribute positively to auditory function. Specifically, we observed superior auditory results in higher frequencies in patients who underwent mastoidectomy promptly following AM diagnosis, highlighting the potential advantage of early surgical intervention in reducing long-term auditory damage associated with severe and prolonged inflammation. This suggests that early mastoidectomy in AM treatment not only has minimal detrimental effects on hearing but may actively promote improved auditory outcomes. While acoustic trauma during mastoidectomy remains a recognized potential risk, primarily due to ultrasonic drill noise, which can generate high-intensity sound exposure potentially affecting high-frequency auditory sensitivity [58,59,60,61,62], the comparable patterns of hearing loss between groups T and TM reinforce the notion that inflammatory processes, rather than surgical intervention, predominantly contribute to observed auditory deficits. The consistency of audiometric findings across both surgical and non-surgical groups further supports the conclusion that observed hearing deficits primarily reflect inflammatory severity and associated physiological changes in middle and inner ear structures.

COMQ-12 scores were generally low, suggesting minimal or negligible chronic otitis media symptoms among study participants.

However, despite these overall low scores, statistically significant differences were noted between the mastoidectomy (TM) and control groups regarding specific symptoms, including hearing difficulties in quiet and noisy environments, discomfort around the ear, sensations of dizziness or vertigo, and tinnitus. These findings indicate that, although most patients experienced only minor symptoms, a subgroup experienced meaningful chronic symptoms possibly impacting their quality of life. This is further supported by a statistically significant correlation between high-frequency hearing loss and higher scores on questions related to hearing in quiet environments, hearing in noise, and tinnitus. A limitation of this assessment is that the questionnaire lacks disease specificity, as acute mastoiditis is an acute rather than a chronic condition; however, instruments designed for acute otitis media (e.g., the Acute Otitis Media Severity of Symptom Scale [63]) were considered less relevant because the patients were not in the acute phase of the disease.

Additionally, sensory loss following surgery was documented in up to 10% of patients, primarily manifesting as diminished tactile sensation around the auricle, which aligns with previously published data [22,64,65]. Otomicroscopic examination further revealed notable structural changes, including tympanic membrane atrophy, myringosclerosis, and epitympanic retractions, clearly associated with inflammatory processes rather than the mastoidectomy itself. The absence of significant differences in structural alterations between groups undergoing tympanostomy alone and those undergoing combined mastoidectomy and tympanostomy suggests these changes result primarily from inflammatory sequelae rather than surgical intervention.

Objective ear function testing (DPOAE, impedance measurements) showed no significant differences attributable to mastoidectomy except elevated stapedius reflex thresholds in the TM group. DPOAE results demonstrate a similar pattern to those observed in pure-tone audiometry. Since tests of inner ear function (pure-tone audiometry, DPOAE) did not show statistically significant differences, we hypothesize that the altered stapedius reflex thresholds reflect biomechanical changes at the level of the middle ear. Additionally, all groups demonstrated lower median middle ear resonance frequencies than the established population averages (0.8–1.2 kHz). The resonance frequency in the control group C was 754 Hz, nearly within the population average, whereas it was lower in groups T (722 Hz) and TM (667 Hz), suggesting some damping due to a mass effect in the affected middle ears [66]. We observed a statistically significant reduction in external auditory canal volume in groups T and TM compared with controls. The underlying cause remains uncertain and could plausibly be attributed to postoperative scarring of the external auditory canal or to alterations in temporal bone pneumatization following infection [67]. Nevertheless, its validity as an objective parameter is questionable, and the clinical significance remains undetermined.

We observed that all patients, except for one with a minor defect, had fully healed cortical bone of the mastoid. This finding indicates that children undergoing mastoidectomy for AM experience a nearly complete restoration of the original anatomical state of the cortical mastoid bone, likely contributing to the preservation of ear function.

The primary goal of the proposed mastoid function test protocol was to assess the suitability and validity of the test. As shown in Table 6, all measurements, except for the temperature change over the mastoid between the 6th and 12th minutes, demonstrated statistically significant differences. The lack of significant temperature change over the mastoid during this interval, despite continued cooling, is understandable as the maximum achievable surface cooling was reached within the first five minutes. Following this period, an increase in local blood flow likely occurred, introducing warmth into the cooled area, while the hydrogel pad itself gradually warmed. Cooling with the hydrogel pad can be conceptualized as point cooling, with surrounding tissues subsequently reheating the cooled area from all directions. Conversely, temperature measurements in the ear canal continued to decrease during the 6–12 min interval, indicating sustained cooling of the deeper temporal (tympanal) bone due to persistent lower surface temperature, an observation consistent with previous studies [26].

Our results indicate that the cooling-based mastoid function test protocol elicited expected and measurable changes in TPP and VORg. However, as this is an experimental method some limitations do apply. Firstly, for routine clinical application validation with a larger sample of healthy subjects is necessary. A larger dataset from healthy individuals would establish a frame Our results indicate that the cooling-based mastoid function test protocol elicited the expected and measurable changes in TPP and VORg. However, as this is an experimental method, several limitations should be noted. First, validation with a larger sample of healthy subjects is required for routine clinical application. Such data would help establish normative reference values against which pathological middle ear conditions (e.g., Eustachian tube dysfunction, serous otitis media, or chronic middle ear infections) could be compared. Second, some concerns regarding reproducibility in daily practice remain, particularly with the cooling procedure. Factors such as the location and timing of application, as well as pad temperature, may vary. Development of an automated cooling device, such as one based on a Peltier thermoelectric module, could standardize this process. Other measurements (e.g., vHIT, tympanometry) are already standardized and should not present difficulties.

Comparison of temperature changes between groups C and TM revealed a roughly linear decrease in ear canal temperatures for both groups, albeit at a slower rate during the second half of the test (Figure 8, Table 6 and Table 7). There were no statistically significant differences between groups concerning the changes in ear canal temperatures. However, statistically significant differences were noted in absolute measurements of mastoid surface temperature at 0, 6, and 12 min, absolute ear canal temperature at 0 min, and the change in mastoid surface temperature between 0–6 and 0–12 min. It should be emphasized that skin surface temperature exhibits substantial individual variability influenced by ambient conditions, limiting its clinical relevance. The significantly greater temperature reduction over the mastoid in the TM group is likely attributed to scar formation and diminished vascularization of the subcutaneous tissues.

Comparison of TPP values during the protocol between groups C and TM showed a faster decrease to slightly lower absolute values in the TM group (Table 7, Figure 9). By the end of the protocol, TPP values in the TM group began to normalize, whereas the control group’s values had not yet started to normalize. None of these differences reached statistical significance. The measured reduction in middle ear pressure during cooling likely results from both air cooling within the middle ear and additional mechanisms, such as changes in mastoid mucosal thickness [26,27,28,68,69]. Since the mastoid’s function as a pressure buffer depends more on volume than on the surface area of its cellular system, and as cortical bone healing post-mastoidectomy closely follows periosteal lines, ultrasound results suggest that mastoid volume remains unchanged. Consequently, we conclude that mastoid pressure regulation capacity is fully preserved following mastoidectomy for AM. Variations in the rate of TPP change could result from altered thermal properties (heat capacity and conductivity) of tissues post-healing compared to healthy tissues.

Direct comparison of absolute VORg values between groups C and TM showed no statistically significant differences (Table 7, Figure 10), nor were there significant differences in VORg changes between the 0–6 min and 6–12 min intervals. However, the overall VORg change (0–12 min) was statistically significant, suggesting a slightly reduced thermal insulation capability of the mastoid. Thermal insulation by the mastoid primarily depends on the ratio of cellular surface area to mastoid volume [26,68,70,71]. Surgical interventions typically result in less extensive regeneration of the mastoid cellular system compared to the cortical bone, with the extent of regeneration strongly influenced by patient age at surgery [72,73]. Both our findings and existing literature indicate partial cellular system regeneration occur, though a permanently lowered cellular surface-to-volume ratio remains likely. The extreme temperature stimulation utilized in this test protocol revealed minor reductions in mastoid thermal insulation, although such stimuli are physiologically uncommon and unlikely to occur in daily life, limiting clinical relevance. Additionally, even the subject who showed incomplete cortical bone healing did not demonstrate significant deviations, suggesting minor bone defects do not substantially influence heat transfer. The occurrence of dizziness, nausea, and nystagmus in two participants can likely be attributed to increased thermal conductivity of tissues post-healing.

## 5. Conclusions

Our study is, to date, the first to provide a detailed analysis of the long-term sequelae of mastoidectomy performed for the treatment of AM, and it is unique in directly comparing two different surgical approaches to middle ear infection. In addition, we developed a novel protocol for the non-invasive measurement of both the pressure regulation capacity and thermal insulation function of the mastoid, which, to our knowledge, has not previously been described in the literature.

Based on the collected data, we found that most patients who underwent mastoidectomy for AM exhibited long-term high-frequency hearing loss of up to 10 dB, minor structural changes in the tympanic membrane, and elevated acoustic reflex thresholds. The same findings were observed in the group with fulminant AOM treated solely by tympanostomy. In the context of AM treatment, the consequences of mastoidectomy are therefore negligible compared to those of less invasive surgical approaches. Better hearing preservation was observed in patients who underwent mastoidectomy earlier in the course of AM.

The proposed method for non-invasive mastoid function testing proved feasible for clinical use. The protocol is reproducible and simple enough to be completed, including patient preparation, within the timeframe typically allocated for standard hearing and balance assessments. Our data demonstrate statistically significant changes in TPP and VORg values before and after completion of the protocol, as well as significant differences between the tested groups. Establishing normative population values would require testing a larger cohort of healthy individuals, which would enhance the clinical applicability of the method and allow comparison with various middle ear pathologies.

Extreme thermal stimulation of the mastoid surface resulted in statistically significant differences in lateral semicircular canal function, indicating that the thermal insulation capacity of the mastoid is slightly reduced after surgery. This change is likely due to a permanently reduced ratio between the surface area of the mastoid cellular system and its volume following mastoidectomy. However, such stimulation is non-physiological and unlikely to occur in everyday life, so we believe this finding has limited practical implications.

Reviewing both the literature and our data, we conclude that mastoidectomy appears to be the most effective surgical method for treating acute mastoiditis, with a negligible incidence of surgical complications. Our findings also suggest that, when required in the pediatric population, mastoidectomy is a safe procedure with minimal long-term consequences, reinforcing its role as a reliable surgical option in the management of this condition. Furthermore, we demonstrated that the earlier mastoidectomy is performed in the course of AM, the lower the likelihood of high-frequency hearing loss. The ability to regulate middle ear pressure after mastoidectomy for AM remains fully preserved. The mastoid’s function as a pressure buffer depends primarily on its volume, and the surgical alteration of the mastoid cell system during cortical mastoidectomy does not affect mastoid volume or its pressure regulation capacity.

## Figures and Tables

**Table 1:** COMQ-12 Questionnaire Results: TM, C—groups; U—Mann–Whitney U test statistic; *p*—statistical significance, Q—question.

	Median (IQR)		Mean (St. Dev.)		Min.; Max.				
Q	TM	C	TM	C	TM	C	U	*p*	r_rb_
1	0.0 (Q_1_: 0.0; Q_3_: 0.0)	0.0 (Q_1_: 0.0; Q_3_: 0.0)	0.2 (0.61)	0.0 (0.0)	0; 3	0; 0	467.0	0.34	−0.269
2	0.0 (Q_1_: 0.0; Q_3_: 0.0)	0.0 (Q_1_: 0.0; Q_3_: 0.0)	0.07 (0.37)	0.0 (0.0)	0; 2	0; 0	464.0	0.3	−0.131
3	0.0 (Q_1_: 0.0; Q_3_: 1.0)	0.0 (Q_1_: 0.0; Q_3_: 0.0)	0.5 (1.01)	0.0 (0.0)	0; 4	0; 0	352.0	0.002	−0.393
4	0.0 (Q_1_: 0.0; Q_3_: 2.0)	0.0 (Q_1_: 0.0; Q_3_: 0.0)	0.93 (1.26)	0.0 (0.0)	0; 4	0; 0	288.0	<0.001	−0.499
5	0.0 (Q_1_: 0.0; Q_3_: 0.25)	0.0 (Q_1_: 0.0; Q_3_: 0.0)	0.33 (0.71)	0.0 (0.0)	0; 3	0; 0	368.0	0.004	−0.365
6	0.0 (Q_1_: 0.0; Q_3_: 0.0)	0.0 (Q_1_: 0.0; Q_3_: 0.0)	0.27 (0.69)	0.0 (0.0)	0; 3	0; 0	400.0	0.017	−0.303
7	0.0 (Q_1_: 0.0; Q_3_: 1.0)	0.0 (Q_1_: 0.0; Q_3_: 0.0)	0.47 (0.90)	0.0 (0.0)	0; 3	0; 0	352.0	0.002	−0.393
8	0.0 (Q_1_: 0.0; Q_3_: 0.0)	0.0 (Q_1_: 0.0; Q_3_: 0.0)	0.0 (0.0)	0.0 (0.0)	0; 0	0; 0	480.0	1	/
9	0.0 (Q_1_: 0.0; Q_3_: 0.0)	0.0 (Q_1_: 0.0; Q_3_: 0.0)	0.07 (0.37)	0.0 (0.0)	0; 2	0; 0	464.0	0.3	−0.131
10	0.0 (Q_1_: 0.0; Q_3_: 0.0)	0.0 (Q_1_: 0.0; Q_3_: 0.0)	0.2 (0.76)	0.0 (0.0)	0; 3	0; 0	448.0	0.14	−0.187
11	0.0 (Q_1_: 0.0; Q_3_: 0.0)	0.0 (Q_1_: 0.0; Q_3_: 0.0)	0.07 (0.36)	0.0 (0.0)	0; 2	0; 0	464.0	0.3	−0.131
12	0.0 (Q_1_: 0.0; Q_3_: 0.0)	0.0 (Q_1_: 0.0; Q_3_: 0.0)	0.2 (0.48)	0.0 (0.0)	0; 2	0; 0	450.0	0.17	−0.303

**Table 2:** Otomicroscopic Examination Results: TM, T, C—groups; χ^2^—Chi-square statistic for the Independent Samples Median Test (ISMT); *p*—statistical significance.

	Median (IQR)			Mean (St. Dev.)				
	TM	T	C	TM	T	C	χ^2^	*p*
Atrophy	0.0 (Q_1_: 0.0; Q_3_: 1.0)	0.0 (Q_1_: 0.0; Q_3_: 1.0)	0.0 (Q_1_: 0.0; Q_3_: 0.0)	0.6 (0.68)	0.4 (0.56)	0.0 (0.0)	19.06	<0.001
Thickening and scarring	0.0 (Q_1_: 0.0; Q_3_: 1.0)	0.0 (Q_1_: 0.0; Q_3_: 1.0)	0.0 (Q_1_: 0.0; Q_3_: 0.0)	0.27 (0.69)	0.27 (0.69)	0.0 (0.0)	5.98	0.5
Myringosclerosis	0.0 (Q_1_: 0.0; Q_3_: 0.0)	0.0 (Q_1_: 0.0; Q_3_: 0.0)	0.0 (Q_1_: 0.0; Q_3_: 0.0)	0.63 (0.89)	0.63 (0.89)	0.0 (0.0)	17.32	<0.001
Myringitis	0.0 (Q_1_: 0.0; Q_3_: 0.0)	0.0 (Q_1_: 0.0; Q_3_: 0.0)	0.0 (Q_1_: 0.0; Q_3_: 0.0)	0.03 (0.18)	0.03 (0.18)	0.0 (0.0)	1.09	0.58
Retraction (Toš)	0.0 (Q_1_: 0.0; Q_3_: 0.0)	0.0 (Q_1_: 0.0; Q_3_: 0.0)	0.0 (Q_1_: 0.0; Q_3_: 0.0)	0.43 (0.68)	0.3 (0.54)	0.0 (0.0)	12.36	0.002
Retraction (Sadé)	0.0 (Q_1_: 0.0; Q_3_: 1.0)	0.0 (Q_1_: 0.0; Q_3_: 1.0)	0.0 (Q_1_: 0.0; Q_3_: 0.0)	0.1 (0.4)	0.1 (0.4)	0.0 (0.0)	2.18	0.336

**Table 3:** Pure-Tone Audiometry Results: TM, T, C—groups; χ^2^—Chi-square statistic for the Independent Samples Median Test (ISMT); *p*—statistical significance.

		C	T	TM	χ^2^	*p*	V
LTA (dB)	Median (IQR)	10.0 (Q_1_: 10.0; Q_3_: 11.66)	11.66 (Q_1_: 10.0; Q_3_: 13.75)	13.3 (Q_1_: 10.0; Q_3_: 18.33)	8.06	0.018	0.30
	Min.	10.0	10.0	10.0			
	Max.	16.67	25.0	31.7			
PTA (dB)	Median (IQR)	10.5 (Q_1_: 10.0; Q_3_: 12.75)	10 (Q_1_: 10.0; Q_3_: 12.25)	11.0 (Q_1_: 10.0; Q_3_: 15.5)	2.27	0.32	0.16
	Min.	10.0	10.0	10.0			
	Max.	19.0	21.8	35.0			
HTA (dB)	Median (IQR)	10 (Q_1_: 10.0; Q_3_: 10)	10.0 (Q_1_: 10.0; Q_3_: 13.33)	15.0 (Q_1_: 10.0; Q_3_: 19.17)	21.7	<0.001	0.48
	Min.	10.0	10.0	10.0			
	Max.	13.33	30.0	108.3			
EHTA (dB)	Median (IQR)	10.71 (Q_1_: 10.0; Q_3_: 14.29)	12.5 (Q_1_: 10.0; Q_3_: 19.82)	18.2 (Q_1_: 10.0; Q_3_: 25.71)	9.3	0.01	0.32
	Min.	7.11	9.3	8.6			
	Max.	25.0	43.6	120			

**Table 4:** DPOAE measurement values: LTA—low-tone average, MTA—mid-to-high frequency average, HTA—high-tone average; χ^2^—Chi-square statistic for the Independent Samples Median Test (ISMT); *p*—statistical significance; TM, T, C—groups.

		C	T	TM	χ^2^	*p*
LTA (dB)	Median (IQR)	6.3 (Q_1_: 4.09; Q_3_: 9.76)	4.38 (Q_1_: 2.8; Q_3_: 10.55)	4.86 (Q_1_: −0.97; Q_3_: 8.35)	0.76	0.68
	Min.	−3.05	−4.45	−6.05		
	Max.	17.15	17.35	16.10		
MTA (dB)	Median (IQR)	19.93 (Q_1_: 10.38; Q_3_: 26.49)	18.0 (Q_1_: 11.78; Q_3_: 23.61)	16.51 (Q_1_: 9.83; Q_3_: 24.71)	3.06	0.216
	Min.	10.38	−7.25	−0.8		
	Max.	34.03	32.45	31.47		
HTA (dB)	Median (IQR)	17.39 (Q_1_: 14.29; Q_3_: 20.58)	10.08 (Q_1_: 5.48; Q_3_: 16.56)	9.36 (Q_1_: 3.99; Q_3_: 12.24)	20.23	<0.001
	Min.	1.8	−1.1	−2.62		
	Max.	30.02	23.95	12.24		

**Table 5:** Thickness of skin over the mastoid and mastoid cortical bone: U—Mann–Whitney U test statistic; *p*—statistical significance; TM, C—groups.

		C	TM	U	*p*
Skin thickness (mm)	Median (IQR)	3.73 (Q_1_: 3.6; Q_3_: 3.93)	3.7 (Q_1_: 3.59; Q_3_: 3.89)	477.0	0.929
	Min.	3.0	3.3		
	Max.	4.7	4.3		
Cortical bone thickness (mm)	Median (IQR)	5.71 (Q_1_: 5.2; Q_3_: 6.36)	5.77 (Q_1_: 5.2; Q_3_: 6.42)	444.0	0.976
	Min.	4.13	1.93		
	Max.	7.97	7.97		
	Min.	1.8	−2.62		
	Max.	30.02	12.24		

**Table 6:** Measurements of ear canal temperature, mastoid skin temperature, VORg, and TPP: C, TM—groups, T_C_—temperature in the ear canal, T_M_—temperature over mastoid, TPP—tympanometric peak pressure, VORg—vestibulo-ocular reflex gain.

		0 Min		6 Min		12 Min	
		C	TM	C	TM	C	TM
T_C_ (°C)	Median (IQR)	36.7 (Q_1_: 36.6; Q_3_: 36.8)	36.6 (Q_1_: 36.4; Q_3_: 36.7)	36.1 (Q_1_: 35.5; Q_3_: 36.3)	35.8 (Q_1_: 35.3; Q_3_: 36.2)	35.75 (Q_1_: 35.1; Q_3_: 36.1)	35.5 (Q_1_: 34.4; Q_3_: 35.9)
	Min.	36.1	36.1	36.1	35.3	33.5	32.7
	Max.	36.9	37.3	36.9	36.2	36.2	36.6
T_M_ (°C)	Median (IQR)	37.3 (Q_1_: 36.9; Q_3_: 37.6)	36.9 (Q_1_: 36.8; Q_3_: 37.1)	18.1 (Q_1_: 16.5; Q_3_: 21.3)	14.8 (Q_1_: 11.9; Q_3_: 20.3)	17.65 (Q_1_: 15.4; Q_3_: 20.9)	15.2 (Q_1_: 13.2; Q_3_: 20.7)
	Min.	33.2	33.7	9.9	8.2	11.2	6.1
	Max.	37.7	37.4	27.5	26.0	28.6	23.3
VORg	Median (IQR)	0.89 (Q_1_: 0.80; Q_3_: 0.96)	0.88 (Q_1_: 0.54; Q_3_: 1.24)	0.93 (Q_1_: 0.86; Q_3_: 1.06)	0.93 (Q_1_: 0.90; Q_3_: 1.06)	1.00 (Q_1_: 0.95; Q_3_: 1.08)	1.06 (Q_1_: 0.95; Q_3_: 1.13)
	Min.	0.66	0.81	0.76	0.80	0.81	0.91
	Max.	1.26	0.95	1.31	1.41	1.36	1.33
TPP (daPa)	Median (IQR)	−1.0 (Q_1_: −5; Q_3_: 0)	−5.0 (Q_1_: −23; Q_3_: 0)	−69.0 (Q_1_: −124; Q_3_: −31)	−89.5 (Q_1_: −65.3; Q_3_: −144.3)	−87.5 (Q_1_: −133; Q_3_: −53.8)	−82.0 (Q_1_: −130.5; Q_3_: −51.3)
	Min.	−75	−135	−216	−296	−208	−241
	Max.	11	15	0	−19	−7	−20

**Table 7:** Comparison of mastoid function measurements between groups (U—Mann–Whitney U test, T_C_—temperature in the ear canal, T_M_—temperature over mastoid, TM, C—groups).

	C	TM	U	*p*
T_M_ 0 min (°C)	37.3 (Q_1_: 36.9; Q_3_: 37.6)	36.9 (Q_1_: 36.8; Q_3_: 37.1)	260	0.002
T_M_ 6 min (°C)	18.1 (Q_1_: 16.5; Q_3_: 21.3)	14.8 (Q_1_: 11.9; Q_3_: 20.3)	317.5	0.022
T_M_ 12 min (°C)	17.65 (Q_1_: 15.4; Q_3_: 20.9)	15.2 (Q_1_: 13.2; Q_3_: 20.7)	302	0.030
T_C_ 0 min (°C)	36.7 (Q_1_: 36.6; Q_3_: 36.8)	36.6 (Q_1_: 36.4; Q_3_: 36.7)	323	0.025
T_C_ 6 min (°C)	36.1 (Q_1_: 35.5; Q_3_: 36.3)	35.8 (Q_1_: 35.3; Q_3_: 36.2)	395	0.230
T_C_ 12 min (°C)	35.75 (Q_1_: 35.1; Q_3_: 36.1)	35.5 (Q_1_: 34.4; Q_3_: 35.9)	359.5	0.189
ΔT_M_ (0–6 min) (%)	−106.6 (Q_1_: −126; Q_3_: −76)	−146.2 (Q_1_: −212; Q_3_: −82)	330	0.035
ΔT_M_ (6–12 min) (%)	1.1 (Q_1_: −24.3; Q_3_: 10.4)	2.9 (Q_1_: 14.2; Q_3_: −12.8)	409.5	0.568
ΔT_M_ (0–12 min) (%)	−103.6 (Q_1_: −141.7; Q_3_: −79.1)	−141.6 (Q_1_: −176.4; Q_3_: −79.8)	313	0.045
ΔT_C_ (0–6 min) (%)	−1.66 (Q_1_: −3.3; Q_3_: −1.37)	−1.8 (Q_1_: −3.6; Q_3_: −1.1)	462	0.8
ΔT_C_ (6–12 min) (%)	−1.11 (Q_1_: −1.42; Q_3_: −0.83)	−1.13 (Q_1_: −0.13; Q_3_: −1.9)	436	0.859
ΔT_C_ (0–12 min) (%)	−2.78 (Q_1_: −4.7; Q_3_: −2.01)	−2.7 (Q_1_: −5.5; Q_3_: −2.2)	423	0.711
VORg (0 min)	0.89 (Q_1_: 0.80; Q_3_: 0.96)	0.88 (Q_1_: 0.54; Q_3_: 1.24)	450.5	0.677
VORg (6 min)	0.93 (Q_1_: 0.86; Q_3_: 1.06)	0.93 (Q_1_: 0.90; Q_3_: 1.06)	453	0.703
VORg (12 min)	1.00 (Q_1_: 0.95; Q_3_: 1.08)	1.06 (Q_1_: 0.95; Q_3_: 1.13)	377	0.292
TPP (0 min) (daPa)	−1.0 (Q_1_: −5; Q_3_: 0)	−5.0 (Q_1_: −23; Q_3_: 0)	342.5	0.051
TPP (6 min) (daPa)	−69.0 (Q_1_: −124; Q_3_: −31)	−89.5 (Q_1_: −65.3; Q_3_: −144.3)	357	0.083
TPP (12 min) (daPa)	−87.5 (Q_1_: −133; Q_3_: −53.8)	−82.0 (Q_1_: −130.5; Q_3_: −51.3)	442	0.929
ΔVORg (0–6 min)	0.06 (Q_1_: 0.032; Q_3_: 0.08)	0.085 (Q_1_: 0.03; Q_3_: 0.12)	378	0.152
ΔVORg (6–12 min)	0.045 (Q_1_: 0.023; Q_3_: 0.11)	0.055 (Q_1_: 0.22; Q_3_: 0.11)	386	0.357
ΔVORg (0–12 min)	0.10 (Q_1_: 0.09; Q_3_: 0.013)	0.14 (Q_1_: 0.10; Q_3_: 0.18)	304	0.033
ΔTPP (0–6 min) (daPa)	−66.5 (Q_1_: −113.5; Q_3_: −27.8)	−72.5 (Q_1_: −127.8; Q_3_: −50)	403.5	0.281
ΔTPP (6–12 min) (daPa)	−21 (Q_1_: −33; Q_3_: −22.8)	−4 (Q_1_: −35; Q_3_: 45.3)	339.5	0.108
ΔTPP (0–12 min) (daPa)	−87 (Q_1_: −121; Q_3_: −51.5)	−71.5 (Q_1_: −101.8; Q_3_: −39.3)	388.5	0.378

## Data Availability

The data presented in this study are available on request from the corresponding author due to privacy issues.

## Abbreviations

The following abbreviations are used in this manuscript:

AM	Acute mastoiditis
AOM	Acute otitis media
COMQ-12	Chronic Otitis Media Questionnaire 12
daPa	Deka pascal
DPOAE	Distortion-Product Otoacoustic Emissions
K	Kelvin
m	Meter
TPP	tympanometric peak pressure
VORg	vestibulo-ocular reflex gain
vHIT	video head impulse test
W	Watt

## References

[1] Luntz M., Brodsky A., Nusem S., Kronenberg J., Keren G., Migirov L., Cohen D., Zohar S., Shapira A., Ophir D. (2001). Acute mastoiditis—The antibiotic era: A multicenter study. Int. J. Pediatr. Otorhinolaryngol..

[2] Zanetti D., Nassif N. (2006). Indications for surgery in acute mastoiditis and their complications in children. Int. J. Pediatr. Otorhinolaryngol..

[3] Kværner K.J., Bentdal Y., Karevold G. (2007). Acute mastoiditis in Norway: No evidence for an increase. Int. J. Pediatr. Otorhinolaryngol..

[4] Psarommatis I.M., Voudouris C., Douros K., Giannakopoulos P., Bairamis T., Carabinos C. (2012). Algorithmic management of pediatric acute mastoiditis. Int. J. Pediatr. Otorhinolaryngol..

[5] Pang L.H.Y., Barakate M.S., Havas T.E. (2009). Mastoiditis in a paediatric population: A review of 11 years experience in management. Int. J. Pediatr. Otorhinolaryngol..

[6] Lin H.W., Shargorodsky J., Gopen Q. (2010). Clinical Strategies for the Management of Acute Mastoiditis in the Pediatric Population. Clin. Pediatr..

[7] Hullegie S., Venekamp R.P., Van Dongen T.M.A., Hay A.D., Moore M.V., Little P., Schilder A.G.M., Damoiseaux R.A.M.J. (2021). Prevalence and Antimicrobial Resistance of Bacteria in Children with Acute Otitis Media and Ear Discharge: A Systematic Review. Pediatr. Infect. Dis. J..

[8] Geva A., Oestreicher-Kedem Y., Fishman G., Landsberg R., DeRowe A. (2008). Conservative management of acute mastoiditis in children. Int. J. Pediatr. Otorhinolaryngol..

[9] Chesney J., Black A., Choo D. (2014). What is the best practice for acute mastoiditis in children?. Laryngoscope.

[10] Bakhos D., Trijolet J.-P., Morinière S., Pondaven S., Al zahrani M., Lescanne E. (2011). Conservative Management of Acute Mastoiditis in Children. Arch. Otolaryngol. Head Neck Surg..

[11] Psarommatis I., Giannakopoulos P., Theodorou E., Voudouris C., Carabinos C., Tsakanikos M. (2012). Mastoid subperiosteal abscess in children: Drainage or mastoidectomy?. J. Laryngol. Otol..

[12] Andersen S.A.W., Mikkelsen P.T., Konge L., Cayé-Thomasen P., Sørensen M.S. (2016). Cognitive load in distributed and massed practice in virtual reality mastoidectomy simulation. Laryngoscope.

[13] Attlmayr B., Zaman S., Scott J., Derbyshire S.G., Clarke R.W., De S. (2015). Paediatric acute mastoiditis, then and now: Is it more of a problem now?. J. Laryngol. Otol..

[14] Nicastro V., Zagaria A., Abita P., Alberti G., Loteta S., Azieli C. (2019). Association between obstructive sleep apnea and hearing loss: A literary review. Acta Medica Mediterr..

[15] Glynn F., Osman L., Colreavy M., Rowley H., Dwyer T.P.O., Blayney A. (2008). Acute mastoiditis in children: Presentation and long term consequences. J. Laryngol. Otol..

[16] Guillén-Lozada E., Bartolomé-Benito M., Moreno-Juara Á. (2023). Surgical management of mastoiditis with intratemporal and intracranial complications in children. Outcome, complications, and predictive factors. Int. J. Pediatr. Otorhinolaryngol..

[17] Harley E.H., Sdralis T., Berkowitz R.G. (1997). Acute Mastoiditis in Children: A 12-Year Retrospective Study. Otolaryngol.–Head Neck Surg..

[18] Petersen C.G., Ovesen T., Pedersen C.B. (2000). Acute mastoidectomy in a Danish county from 1977 to 1997-operative findings and long-term results. Acta Oto-Laryngol. Suppl..

[19] Enoksson F., Groth A., Hultcrantz M., Stalfors J., Stenfeldt K., Hermansson A. (2015). Subperiosteal abscesses in acute mastoiditis in 115 Swedish children. Int. J. Pediatr. Otorhinolaryngol..

[20] Tos M., Poulsen G. (1980). Attic Retractions Following Secretory Otitis. Acta Oto-Laryngol..

[21] Sade J., Berco E. (1976). Atelectasis and Secretory Otitis Media. Ann. Otol. Rhinol. Laryngol..

[22] Kang H.S., Ahn S.K., Jeon S.Y., Hur D.G., Kim J.P., Park J.J., Kim D.W., Woo S.H. (2012). Sensation recovery of auricle following chronic ear surgery by retroauricular incision. Eur. Arch. Oto-Rhino-Laryngol..

[23] Phillips J.S., Haggard M., Yung M. (2014). A new health-related quality of life measure for active chronic otitis media (COMQ-12): Development and initial validation. Otol. Neurotol..

[24] Vozel D., Steiner N., Božanić Urbančič N., Mladenov D., Battelino S. (2020). Slovenian Cross-Cultural Adaptation and Validation of Health-Related Quality of Life Measures for Chronic Otitis Media (COMQ-12), Vertigo (DHI, NVI) and TINNITUS (THI). Zdr. Varst..

[25] Hearing C.O., Balkany T.A., Gates G.A., Goldenberg R.A., Meyerhoff W.L., House J.W., Surg O.H.N. (1995). Committee on Hearing and Equilibrium guidelines for the evaluation of results of treatment of conductive hearing loss. Otolaryngology-Head Neck Surg..

[26] Magnuson B. (2003). Functions of the mastoid cell system: Auto-regulation of temperature and gas pressure. J. Laryngol. Otol..

[27] Fooken Jensen P.V., Gaihede M. (2016). Congestion of mastoid mucosa and influence on middle ear pressure—Effect of retroauricular injection of adrenaline. Hear. Res..

[28] Cros O., Borga M., Pauwels E., Dirckx J.J.J., Gaihede M. (2013). Micro-channels in the mastoid anatomy. Indications of a separate blood supply of the air cell system mucosa by micro-CT scanning. Hear. Res..

[29] Suzuki M., Kadir A., Hayashi N., Takamoto M. (1998). Direct influence of temperature on the semicircular canal receptor. J. Vestib. Res. Equilib. Orientat..

[30] Smolders J.W.T., Klinke R. (1984). Effects of temperature on the properties of primary auditory fibres of the spectacled caiman, *Caiman crocodilus* (L.). J. Comp. Physiol. A.

[31] Whitehead M.L., Wilson J.P., Baker R.J. (1986). The Effects of Temperature on Otoacoustic Emission Tuning Properties. Auditory Frequency Selectivity.

[32] Ferber-Viart C., Savourey G., Garcia C., Duclaux R., Bittel J., Collet J. (1995). Influence of hyperthermia on cochlear micromechanical properties in humans. Hear. Res..

[33] Zenner H.P., Zimmermann U. (1995). Caloric evoked motile responses of mammalian vestibular sensory cells. Acta Oto-Laryngol..

[34] Ohtani M., Yamashita T., Amano H., Kubo N., Kumazawa T. (1993). Thermal influence on intracellular calcium concentration in vestibular hair cells isolated from the guinea pig. A preliminary report. Acta Oto-Laryngol. Suppl..

[35] Hood J.D. (1989). Evidence of direct thermal action upon the vestibular receptors in the caloric test. A re-interpretation of the data of Coats and Smith. Acta Otolaryngol..

[36] Feldmann A., Wili P., Maquer G., Zysset P. (2018). The thermal conductivity of cortical and cancellous bone. Eur. Cells Mater..

[37] Xu F., Lu T.J., Seffen K.A., Ng E.Y.K. (2009). Mathematical modeling of skin bioheat transfer. Appl. Mech. Rev..

[38] Rojas-Altamirano G., Vargas R.O., Escandón J.P., Mil-Martínez R., Rojas-Montero A. (2022). Calculation of Effective Thermal Conductivity for Human Skin Using the Fractal Monte Carlo Method. Micromachines.

[39] Ni Y., Sha Y., Dai P., Li H. (2008). Quantitative morphology of facial nerve based on three-dimensional reconstruction of temporal bone. Otolaryngol. Head Neck Surg..

[40] Merati M., Kazemi M.A., Dabiri S., Kouhi A. (2021). Radiologic evaluation of the mastoid segment of the facial nerve tract in the intact temporal bone. Surg. Radiol. Anat..

[41] Mahboubi H., Wu E.C., Jahanbakhshi R., Coale K., Rothholtz V.S., Zardouz S., Djalilian H.R. (2012). A novel method to determine standardized anatomic dimensions of the osseous external auditory canal. Otol. Neurotol..

[42] Palva T., Virtanen H., Mäkinen J. (1985). Acute and latent mastoiditis in children. J. Laryngol. Otol..

[43] Van Zuijlen D.A., Schilder A.G.M., Van Balen F.A.M., Hoes A.W. (2001). National differences in incidence of acute mastoiditis: Relationship to prescribing patterns of antibiotics for acute otitis media?. Pediatr. Infect. Dis. J..

[44] Bento R.F., Fonseca ACde O. (2013). A brief history of mastoidectomy. Int. Arch. Otorhinolaryngol..

[45] Bartov N., Lahav Y., Lahav G., Zloczower E., Katzenell U., Halperin D., Hilly O., Shoffel-Havakuk H. (2019). Management of Acute Mastoiditis With Immediate Needle Aspiration for Subperiosteal Abscess. Otol. Neurotol..

[46] Spratley J., Silveira H., Alvarez I., Pais-Clemente M. (2000). Acute mastoiditis in children: Review of the current status. Int. J. Pediatr. Otorhinolaryngol..

[47] Cohen-Kerem R., Uri N., Rennert H., Peled N., Greenberg E., Efrat M. (1999). Acute mastoiditis in children: Is surgical treatment necessary?. J. Laryngol. Otol..

[48] Palva T., Ramsay H. (1996). Incudal Folds and Epitympanic Aeration. Otol. Neurotol..

[49] Groth A., Enoksson F., Hultcrantz M., Stalfors J., Stenfeldt K., Hermansson A. (2012). Acute mastoiditis in children aged 0–16 years—A national study of 678 cases in Sweden comparing different age groups. Int. J. Pediatr. Otorhinolaryngol..

[50] Stalfors J., Enoksson F., Hermansson A., Hultcrantz M., Robinson Å., Stenfeldt K., Groth A. (2013). National assessment of validity of coding of acute mastoiditis: A standardised reassessment of 1966 records. Clin. Otolaryngol..

[51] Enoksson F. (2015). Acute Mastoiditis in Children—A National Study in Sweden. Ph.D. Thesis.

[52] Lee H.J., Woo J.H., Cho S., Oh H.W., Joo H., Baik H.J. (2020). Risk Factors for Perioperative Respiratory Adverse Events in Children with Recent Upper Respiratory Tract Infection: A Single-Center-Based Retrospective Study. Ther. Clin. Risk Manag..

[53] Groth A., Enoksson F., Stalfors J., Stenfeldt K., Hultcrantz M., Hermansson A. (2012). Recurrent acute mastoiditis—A retrospective national study in Sweden. Acta Oto-Laryngol..

[54] Schwam Z.G., Michaelides E., Kuo P., Hajek M.A., Judson B.L., Schutt C. (2018). Thirty-day morbidity and mortality following otologic/neurotologic surgery: Analysis of the national surgical quality improvement program. Laryngoscope.

[55] Prinsley P. (2013). An audit of “dead ear” after ear surgery. J. Laryngol. Otol..

[56] Tait A.R., Malviya S., Voepel-Lewis T., Munro H.M., Seiwert M., Pandit U.A. (2001). Risk factors for perioperative adverse respiratory events in children with upper respiratory tract infections. Anesthesiology.

[57] Michel F., Vacher T., Julien-Marsollier F., Dadure C., Aubineau J.-V., Lejus C., Sabourdin N., Woodey E., Orliaguet G., Brasher C. (2018). Peri-operative respiratory adverse events in children with upper respiratory tract infections allowed to proceed with anaesthesia: A French national cohort study. Eur. J. Anaesthesiol..

[58] Yin X., Strömberg A.-K., Duan M. (2011). Evaluation of the noise generated by otological electrical drills and suction during cadaver surgery. Acta Otolaryngol..

[59] Michaelides E.M., Kartush J.M. (2001). Implications of sound levels generated by otologic devices. Otolaryngol. Head Neck Surg..

[60] Maccà I., Scapellato M.L., Carrieri M., Maso S., Trevisan A., Bartolucci G.B. (2015). High-frequency hearing thresholds: Effects of age, occupational ultrasound and noise exposure. Int. Arch. Occup. Environ. Health.

[61] Stenfelt S., Goode R.L. (2005). Transmission properties of bone conducted sound: Measurements in cadaver heads. J. Acoust. Soc. Am..

[62] Abtahi S.H., Fazel A., Rogha M., Nilforoush M., Solooki R. (2016). Effect of drill-induced noise on hearing in non-operated ear. Adv. Biomed. Res..

[63] Shaikh N., Rockette H.E., Hoberman A., Kurs-Lasky M., Paradise J.L. (2015). Determination of the Minimal Important Difference for the Acute Otitis Media Severity of Symptom Scale. Pediatr. Infect. Dis. J..

[64] Frampton S.J., Pringle M. (2011). Cutaneous sensory deficit following post-auricular incision. J. Laryngol. Otol..

[65] Vakharia S.D., Gupta S.R. (2022). Sensation Loss of Auricle Following Ear Surgery by Post-auricular Incision: Our Experience. Indian J. Otolaryngol. Head Neck Surg..

[66] Zloczower E., Tsur N., Hershkovich S., Fink N., Marom T. (2022). Efficacy of Oral Steroids for Acute Acoustic Trauma. Audiol. Neurootol..

[67] Cayé-Thomasen P., Hermansson A., Tos M., Prellner K. (1999). Bone modeling dynamics in acute otitis media. Laryngoscope.

[68] Swarts J.D., Cullen Doyle B.M., Alper C.M., Doyle W.J. (2010). Surface area-volume relationships for the mastoid air cell system and tympanum in adult humans: Implications for mastoid function. Acta Oto-Laryngol..

[69] Gaihede M. (2000). Middle ear volume and pressure effects on tympanometric middle ear pressure determination: Model experiments with special reference to secretory otitis media. Auris Nasus Larynx.

[70] Cros O., Knutsson H., Andersson M., Pawels E., Borga M., Gaihede M. (2016). Determination of the mastoid surface area and volume based on micro-CT scanning of human temporal bones. Geometrical parameters depend on scanning resolutions. Hear. Res..

[71] Lima M.A.R., Farage L., Cury M.C.L., Júnior F.B. (2013). Mastoid surface area-to-volume ratios in adult brazilian individuals. Braz. J. Otorhinolaryngol..

[72] Kwon O.J., Sung J.M., Jung H.K., Kim C.W. (2017). Postoperative Mastoid Aeration Following Canal Wall Up Mastoidectomy according to Preoperative Middle Ear Disease: Analysis of Temporal Bone Computed Tomography Scans. J. Audiol. Otol..

[73] Kaneko K., Kanemaru S., Kanai R., Atsushi Y. (2012). Regeneration of Mastoid Air Cells in Vivo Using Autologous Cortical Bone. Surg. Sci..

